# Nitrous Oxide-Induced Subacute Combined Degeneration in a 38-Year-Old Pregnant Female After Recreational Use

**DOI:** 10.7759/cureus.37696

**Published:** 2023-04-17

**Authors:** Joshua R Jones, Skyler Porcaro, Nelson Jones, Gagandeep Gill, Erwin Patalinghug

**Affiliations:** 1 Anesthesiology, Oakland University William Beaumont School of Medicine, Rochester, USA; 2 Physical Medicine and Rehabilitation, Oakland University William Beaumont School of Medicine, Rochester, USA; 3 Anesthesiology, Brigham Young University, Provo, USA; 4 Family Medicine, Beaumont Hospital, Troy, USA

**Keywords:** pregnancy, neuropathy, b12 deficiency, nitrous oxide inhalation, nitrous oxide abuse, nitrous oxide toxicity, laughing gas, subacute combined degeneration, nitrous oxide

## Abstract

Nitrous oxide (N2O) misuse creates a diagnostic dilemma due to its clinical presentation, difficulty in identification, and toxicity related to its chronic abuse, with resultant morbidity and mortality. Chronic abuse can lead to myeloneuropathy and subacute combined degeneration in otherwise healthy individuals. Health professionals should be aware of the commercial availability and abuse of N2O by the public, and N2O toxicity should be included in the differential diagnosis in patients presenting with myelopathy of unknown etiology. A case report was conducted on a 38-year-old female at approximately 30 weeks of gestation who presented to the emergency department with worsening bilateral lower extremity numbness, tingling, and weakness. The patient admitted to nitrous oxide inhalation during the two months prior to admission. She reported using four cans of whippets per week (approximately 8 g of N2O per whippet) up to 50 cans per day (400 g N2O) prior to the onset of symptoms. An MRI of the cervical spine was performed, showing T2 hyperintensity from C2 to C6 involving dorsal columns indicative of subacute combined degeneration. The patient was treated with intravenous vitamin B12 due to the clinical and radiographic evidence of nitrous oxide-induced myelopathy. The pathophysiology of N2O toxicity involves the oxidation of the cobalt atom of cobalamin (vitamin B12) from its reduced active 1+ valent state to its oxidized inactive 3+ valent state. This oxidation inactivates the enzyme methionine synthetase. B12 is an essential cofactor for downstream DNA synthesis. Consequently, excess N2O creates functional B12 deficiency leading to irreversible nerve damage if left undiagnosed and untreated.

## Introduction

Nitrous oxide (N2O) is a colorless, odorless, non-flammable gas commonly used in medical/dental procedures for its analgesic and anesthetic properties. The gas is also used as a propellant in whipped cream canisters (whippets) and a power booster in automobiles and motorcycles. Unfortunately, the drug has also become increasingly popular as an inhalant in the psychedelic community in the United States [1,2]. The drug is abused commonly through the use of whippets, which are readily available for purchase online and at most supermarkets and convenience stores. The end of the small whippet canister is pierced, and the escaping gas is captured in a balloon and then subsequently inhaled. The average whippet canister contains approximately 8 g of N2O. The desired effects of recreational use include euphoria, analgesia, lightheadedness, and a general state of intoxication. The effects of N2O usually only last for a short period of time (15-30 minutes); however, a sustained effect can be achieved through continuous/repeated use. Neurologic (ataxia, neuropathy, psychosis), hematologic (B12 deficiency), and reproductive (fetotoxicity, DNA damage) side effects and toxicity have been documented among those who abuse nitrous oxide and even among healthcare workers [3-5]. This case report describes a 38-year-old G4P2012 Caucasian female at approximately 30 weeks of gestation with no prenatal care who presented with bilateral lower extremity myelopathy/paralysis secondary to a functional vitamin B12 deficiency from N2O abuse.

## Case presentation

A 38-year-old G4P2012 white female at approximately 30 weeks of gestation with no prior prenatal care and with a past medical history significant for Grave’s disease status post partial thyroidectomy presented to the emergency department with two weeks of progressively worsening and ascending bilateral lower extremity numbness, tingling, and weakness. The symptoms had involved her entire lower extremities three days prior to presentation, resulting in difficulties in standing, walking, or even supporting her weight. She also reported worsening numbness and tingling that extended to the mid-forearm and weakness in her upper extremities, with a subsequent reduction in forearm grip strength. The patient denied any history of similar symptoms in the past. She denied any injuries or trauma and a comprehensive review of the systems was only positive for back pain. The patient was seen at a different hospital earlier and was told that her symptoms were due to carpal tunnel and sent home.

Upon arrival at the emergency department, her vitals were stable, and her initial complete metabolic panel, thyroid stimulating hormone, magnesium level, and creatine kinase were within normal limits. A complete blood count demonstrated a low hemoglobin of 10.7 g/dL with an elevated mean corpuscular volume of 104 fL, consistent with macrocytic anemia. Urinalysis was positive for 4+ bacteria, 21-50 white blood cells, and 3+ leukocyte esterase. After consultation with neurology, an MRI of the cervical spine was performed, showing T2 hyperintensity from C2 to C6 that involved the dorsal columns. MRI of the thoracic and lumbar spine was declined by the patient due to discomfort and inability to remain still.

**Figure 1:**
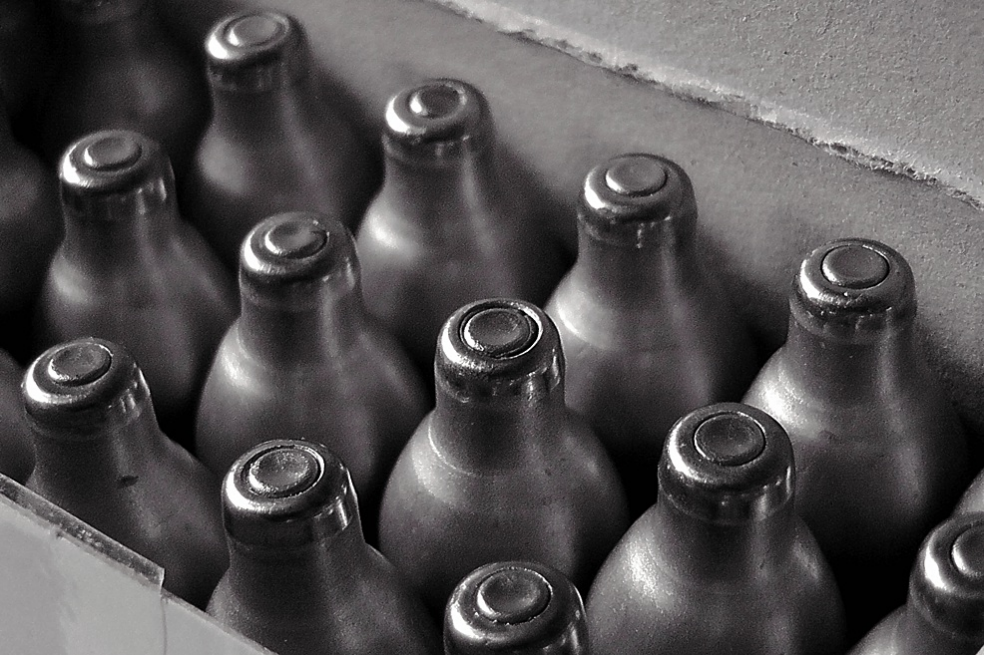
Nitrous oxide (whippet) canisters Image used under CC BY-SA 3.0 from https://commons.wikimedia.org/wiki/File:N2O_whippets.jpg.

The patient admitted to a current half-pack per day smoking history over the last 15 years, as well as a history of recreational drug use including cocaine (with the last episode of inhaling through her nose occurring one week prior to admission), marijuana, benzodiazepines (with a history of benzodiazepine withdrawal), and nitrous oxide inhalation (with the last episode occurring two days prior to admission) but denied alcohol use or IV drug use. The nitrous oxide inhalation had become more frequent over the past two months, increasing from four whippet canisters per week (approximately 8 g of N2O per whippet) to a daily use of 50 whippet canisters (400 g N2O) before the onset of symptoms and admission to the hospital (Figure 1) [6]. A urine drug screen, which was ordered in the emergency department given the patient's stated history, was positive for marijuana and cocaine.

The patient was admitted to the family medicine service due to her lower extremity numbness, tingling, and weakness, as well as the concern for nitrous oxide toxicity. Neurology, physical medicine and rehabilitation, and poison control were consulted, and recommended treatment with a 1000 mcg injection of vitamin B12, due to the radiographic evidence of nitrous oxide-induced myelopathy. Differential considerations for this pattern of spinal cord signal hyperintensity included B12 deficiency, HIV disease, vitamin E, and copper deficiency. Additional considerations, though less likely, were neurosyphilis and sarcoidosis. B12 deficiency and nitrous oxide toxicity were the favored diagnoses.

Because of her gestational status, maternal-fetal medicine was consulted and recommended a transabdominal ultrasound, which revealed a low-lying and posterior placenta. A transvaginal ultrasound was preferred, but unable to be performed due to the patient’s lower extremity weakness and paralysis. Of note, the patient stated that given her plans to put the baby up for adoption, she had not sought prenatal care. She had three previous pregnancies, two of which were completed through spontaneous vaginal delivery, and her third pregnancy was aborted because of benzodiazepine misuse that lead to seizures and withdrawal-like symptoms. Otherwise, she denied a history of hypertension during pregnancy, gestational diabetes, or any other pregnancy complications. In her current pregnancy, she denied any indications of preterm labor, vaginal bleeding, or leakage of fluid. Throughout her admission, she reported good fetal movement.

Further laboratory work was performed during her stay, which showed normal levels of thyroid stimulating hormone (TSH), creatine kinase (CK), erythrocyte sedimentation rate (ESR), and C-reactive protein (CRP), and negative antinuclear antibodies (ANA), negative rapid plasma reagin (RPR), and negative HIV. The following laboratory values came back abnormal: vitamin B12, homocysteine, vitamin B6, 25-hydroxy vitamin D, mean corpuscular volume (MCV), and iron (Fe) (Table 1). Her urine culture was positive for *Escherichia coli*, for which she was treated with nitrofurantoin.

**Table 1:** Patient's abnormal lab values

Lab	Patient value	Normal range
Vitamin B12	<146 pg/mL	271-1000 pg/mL
Homocysteine	>50 µmol/L	4-10 µmol/L
Vitamin B6	3 mcg/L	5-50 mcg/L
25-hydroxy vitamin D	8 ng/mL	30-100 ng/mL
Mean corpuscular volume (MCV)	103 fL	80-100 fL
Iron (Fe)	26 mcg/dL	50-170 mcg/dL

As a result of the lower extremity numbness, tingling, and weakness, gestational status, and fall risk, it was recommended that the patient continue care and receive physical and occupational therapy. Despite needing assistance to perform most activities of daily living, some improvement in her lower extremities now allowed her to sit up, stand, and ambulate with a rolling walker for an average of 20 feet with minimal assistance. However, her sensation to light touch, vibration, and proprioception in both lower extremities remained unchanged since her admission. A subacute rehabilitation facility was recommended to further her physical progress, but these services were declined by the patient. Subsequent home care and home physical therapy services were pursued. Over 70 home care programs were contacted and were either outside of the patient’s service area or did not accept her insurance. After becoming more irritated and aggressive toward the staff, the patient decided that she wanted to leave the hospital. She was made aware of the risks that her diagnosis posed to her health and the risks to her pregnancy, and she received recommendations for outpatient follow-up with family medicine and obstetrics. Upon obtaining a wheelchair, the patient left the hospital against medical advice prior to the resolution of her symptoms.

Upon additional review of the electronic health record, as well as accessible outside records, there was no evidence of the patient receiving any follow-up care, either related to her musculoskeletal complaints or her pregnancy, during the three-and-a-half months following her hospital discharge.

Due to the severity of the patient’s symptoms, the irreversible nature of vitamin B12 deficiency-related myelopathy, the patient’s complex biopsychosocial interactions, and issues related to social determinants of health, including substance use disorder, unemployment, and difficult relationships, it is anticipated that without proper treatment, follow-up, and rehabilitation, the patient will experience an indolent course of recovery with possible long-term, irreversible sequelae.

## Discussion

Due to the severity of the patient’s symptoms, the irreversible nature of vitamin B12 deficiency-related myelopathy, the patient’s complex biopsychosocial interactions, and issues related to social determinants of health, including substance use disorder, unemployment, and difficult relationships, it is anticipated that without proper treatment, follow-up, and rehabilitation, the patient will experience an indolent course of recovery with possible long-term, irreversible sequelae. This case report highlights the need to consider nitrous oxide exposure or abuse when patients present with symptoms of myelopathy or subacute combined degeneration.

Nitrous oxide misuse creates a poignant problem because of the difficulty in identification and the toxicity related to chronic abuse, including possible death [7,8]. When inhaled, nitrous oxide has been documented to have analgesic, anxiolytic, anesthetic, and euphoric properties [9,10]. However, chronic abuse can lead to myeloneuropathy and subacute combined degeneration, neuroimaging changes, psychosis, skin hyperpigmentation, cytopenia, and possible death in otherwise healthy individuals [11,12]. While some of the symptoms mentioned above, such as myeloneuropathy and subacute combined degeneration, are pathognomonic for nitrous oxide misuse, other drugs of abuse may present with varying neurological symptoms and sequelae (Table 2) [13].

**Table 2:** Adverse neurological effects of common drugs of abuse Compiled from the National Institutes of Health (NIH) National Institute on Drug Abuse - Commonly Used Drug Charts.

Common drugs of abuse	Adverse neurological effects
Cannabis (marijuana/pot/weed)	Enhanced sensory perception, euphoria, slowed reaction time, problems with balance and coordination, problems with learning and memory, mental health changes
Central nervous system depressants (benzos)	Drowsiness, slurred speech, poor concentration, confusion, dizziness, problems with movement and memory
Cocaine (coke/crack)	Enlarged pupils, headache, euphoria, alertness, insomnia, anxiety, paranoia, psychosis, seizure, loss of olfaction
Gamma-hydroxybutyrate (GHB)	Euphoria, confusion, memory loss, seizures
Heroin	Euphoria, analgesia
Nitrous Oxide (N2O)	Confusion, slurred speech, lack of coordination, euphoria, dizziness, disinhibition, lightheadedness, headaches, seizures, nerve damage, brain damage
Ketamine	Problems with attention and learning, hallucinations, confusion, unconsciousness, depression, poor memory
Lysergic acid diethylamide (LSD; acid)	Emotional swings, perceptual distortions, dizziness, tremors, enlarged pupils, flashbacks, visual disturbances, disorganized thinking, paranoia
MDMA (ecstasy/molly)	Lowered inhibition, enhanced sensory perception, muscle tension, depression, problems with attention and memory, sleep problems, anxiety
Methamphetamine (crystal/meth)	Increased wakefulness, anxiety, confusion, insomnia, mood problems, paranoia, hallucinations, delusions
Prescription opioids (oxy/percs)	Analgesia, drowsiness, euphoria

The adverse effects of nitrous oxide have been described as the oxidation of the cobalt atom of cobalamin (vitamin B12) from its reduced active 1+ valent state to its inactive 3+ valent state. This oxidation inactivates the enzyme methionine synthetase, which requires vitamin B12 as an essential cofactor for eventual DNA synthesis. In turn, a functional B12 deficiency can precipitate, leading to irreversible nerve damage if left undiagnosed and untreated [14,15]. It is understood that the lack of vitamin B12 induces swelling of the myelin sheath that becomes deformed and irregular, eventually precipitating complete demyelination of the axon [16,17]. The cervical and upper thoracic segments of the spinal cord are typically affected as the lesion spreads laterally and longitudinally. This can include the involvement of the posterior columns, posterior dorsal roots, and posterolateral regions of the spinal cord. Increased water content secondary to edema creates the characteristic hyperintense lesion on T2-weighted MRI scans [11].

**Figure 2:**
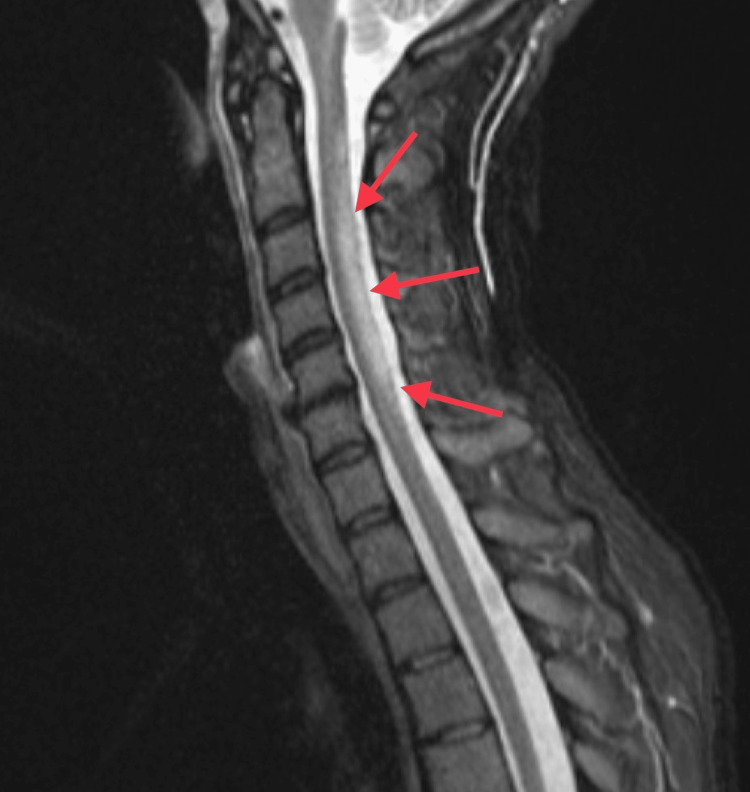
MRI showing T2 hyperintensity involving the dorsal columns of C2-C6

The cervical spine T2-weighted hyperintensity present in our patient’s MRI (Figure 2) is a neuropathological change similar to changes seen in patients with subacute combined degeneration [12,13]. If nitrous oxide-related myeloneuropathy is suspected, treatment can be initiated by administering vitamin B12 supplements [11]. The time it takes for a patient to experience some or any alleviation of symptoms varies. Recovery of neurologic function depends on the duration of abuse and the neuropathological damage sustained to the spinal cord [14,15]. One review of subacute combined degenerations suggested improved neurologic recovery when positive Romberg and positive Babinski signs were absent on the physical exam. Supplementation with methionine has also been reported to arrest the progression of nitrous oxide-induced myelopathy and support recovery [18].

Nitrous oxide is classified as a Category C pregnancy risk medication, meaning that there is a risk of fetal harm if administered during pregnancy. Therefore, it is recommended that pregnant women avoid exposure to nitrous oxide. A 1999 study by Bodin et al. examined the relationship between shift work and occupational nitrous oxide exposure in the second trimester of pregnancy among Swedish midwives. Their findings suggested that exposure to nitrous oxide use was associated with a statistically significant decrease in birth weight and an increase in the odds of being small for gestational age (SGA). Furthermore, a 1995 study by Rowland et al. looked into the relationship between nitrous oxide gas exposure and spontaneous abortion among female dental procedures. They found a statistically significant elevation in the risk of spontaneous abortion (relative risk = 2.6) among those women who worked with nitrous oxide for three or more hours without adequate ventilation systems.

## Conclusions

Clinicians should be aware of the commercial availability and increasing abuse of nitrous oxide by the lay public via the legal purchasing of whippet canisters used for household cream dispensers. Due to the growing prevalence of the recreational use of nitrous oxide, it is prudent that all patients presenting with features of myelopathy of unknown etiology or symptoms related to vitamin B12 deficiency and subacute combined degeneration be questioned about illicit use of nitrous oxide and other recreational and noxious drugs. It is also recommended that all pregnant women be counseled on the harmful effects that nitrous oxide can have on both the mother and fetus.
